# Implementation of Arithmetic and Nonarithmetic Functions on a Label-free and DNA-based Platform

**DOI:** 10.1038/srep34810

**Published:** 2016-10-07

**Authors:** Kun Wang, Mengqi He, Jin Wang, Ronghuan He, Jianhua Wang

**Affiliations:** 1 Department of Chemistry, College of Sciences, Northeastern University, Shenyang 110819, China; 2 Research Center for Analytical Sciences, College of Sciences, Northeastern University, Shenyang 110819, China

## Abstract

A series of complex logic gates were constructed based on graphene oxide and DNA-templated silver nanoclusters to perform both arithmetic and nonarithmetic functions. For the purpose of satisfying the requirements of progressive computational complexity and cost-effectiveness, a label-free and universal platform was developed by integration of various functions, including half adder, half subtractor, multiplexer and demultiplexer. The label-free system avoided laborious modification of biomolecules. The designed DNA-based logic gates can be implemented with readout of near-infrared fluorescence, and exhibit great potential applications in the field of bioimaging as well as disease diagnosis.

The rapid development of electronic computers has brought huge influence on the molecular field, and vice versa. Especially, studies on molecular computing, a molecular-level data processing, have drawn extensive attentions in the field of information technology^1^,^2^. Fabrication of molecular computer seems to be the viable way to break through the limits of conventional electronic computer, and is the easy way to find artificial intelligence, or real intelligence^3^. Since the first report of molecular logic gate by de Silva in 1993, various molecular logic gates have been developed and a new field of molecular computing has been created^4^. Up to now, the fundamental molecular logic gates, such as ‘AND’, ‘OR’, ‘XOR’, and ‘INHIBIT’^5^,^6^,^7^, have been constructed based on synthetic molecules and biomolecules^8^,^9^,^10^. For further advancements in molecular computation, more complicated molecular logic gates are indispensable. The complex logic circuits are the integration of at least two kinds of logic gates with multiple inputs and outputs, such as half adder and half subtractor, full adder and full subtractor, encoder and decoders, and multiplex and demultiplex^11^,^12^,^13^,^14^,^15^. Fan *et al*. constructed the half adder through combining an XOR and an AND gate^16^. Xia and coworks reported a series of logic gates based on redox-modified DNA as signal reporter^14^. Although molecular logic gate has achieved great advancements, it still remains challenges for creation of advanced logic circuits to satisfy the requirements of progressive computational complexity and cost-effectiveness. One limitation results from the simple combination of independent basic logic gates. The desired circuits for advanced logic gates are constructed based on different platforms^16^. Moreover, the multiple outputs signals are generally produced by modified signal reporter, leading to not only high cost but also more complicated operation processes^12^,^17^. Consideration of the previous work limitations, a label-free and universal platform needs to be constructed for the advanced logic gates.

DNA is a promising candidate for the construction of biological devices due to its unique properties, including structural simplicity, convenient synthesis, high flexibility, and predictable behaviour^18^,^19^. DNA has been widely used to construct the advanced logic devices. Most advanced DNA logic devices are composed of dye-labelled or redox-labelled DNA strands^13^,^17^. However, the modification of DNA leads to not only a high cost of running but also potentially more complex processes such as separation and purification^20^. DNA-template silver nanoclusters (AgNCs), a new class of fluorophores, exhibit subnanometre size, high photostability, non-toxicity, and remarkable biocompatibility^21^,^22^,^23^. In comparison with traditional dyes, DNA-template AgNCs can generate brighter signals and are more cost-effective. And more importantly, DNA-template AgNCs overcome the shortcomings of DNA modification and are the best candidate to be the label-free signal reporter.

Graphene oxide (GO) and water-soluble carbon materials have been deeply and intensively investigated in various fields^24^. A number of studies relate to the exploration of GO nanomaterials as functionalized units for the fabrication of bioelectronic devices or biosensors^9^,^15^. Willner’s group has demonstrated that the GO can quench the fluorescence of AgNCs^25^. Based on this theory, a series of logic functions have been achieved by Dong *et al*.^19^. The superior nanomaterials are one of the best choices to be the label-free quencher. However, it is still a challenge to create advanced logic circuits with progressive computational complexity.

We proposed a label-free platform based on GO and DNA-template AgNCs to avoid the DNA modification. For the potential applications, the proposed platform should have the ability to perform multicomponent functions in order to solve the computational complexity. Thus, a series of complex logic gates were constructed based on the same platform and a constant threshold setpoint to perform different type functions, including arithmetic (half adder and half substractor) and nonarithmetic (multiplexer and demultiplexer) functions. In addition, the label-free system avoided laborious modification of biomolecules, and had a powerful multi-processor function. It’s worth noting that the constructed logic circuits can be implemented with output of near-infrared fluorescence and in enzyme-free condition, having a prospective application on bio-imaging and disease diagnosis and therapy^26^,^27^.

## Results

### Operation of Arithmetic Functions

To implement arithmetic functions, half adder (HA) and half subtractor (HS) are in high demand and can construct more advanced computational functions^28^. Hence, the HA and HS logic gates in proof-of-principle experiments are designed on a label-free and universal platform to carry out multiple arithmetic functions. As an important digital signal processor, a half adder, which can implement an additional operation, is composed of parallel XOR and AND logic gates to produce SUM (S) and CARRY (C) digits, respectively^29^. Similarly, a half subtractor can perform a subtraction of two bits. It can be implemented by integrating an XOR and an INHIBIT gate in parallel to generate a DIFFERENCE (D) and a BORROW (B) output, respectively^30^. The fluorescence of AgNCs and N-methylmesoporphyrin IX (NMM) were selected as output signals. The near-infrared-emitting AgNCs were stabilized by the single-stranded DNA (ss-DNA), which was used as the template (Ag-DNA) in the present system. The fluorescent signal of NMM can be enhanced upon the formation of a G-quadruplex/NMM complex^31^. It has been demonstrated that the ss-DNA could be bound to the surface of GO through π-π stacking interaction, which significantly quenched the fluorescent signal of AgNCs, as given in Fig. S2A^25^. Thus, GO is capable of differentiating ss-DNA from double-stranded DNA (ds-DNA) and the G-quadruplex. The Ag-DNA was desorbed from GO when it formed ds-DNA or G-quadruplex structure depending on the interaction among the designed inputs and the platform. Hence, the label-free and universal platform was composed of the Ag-DNA, NMM and GO.

**Figure 1:**
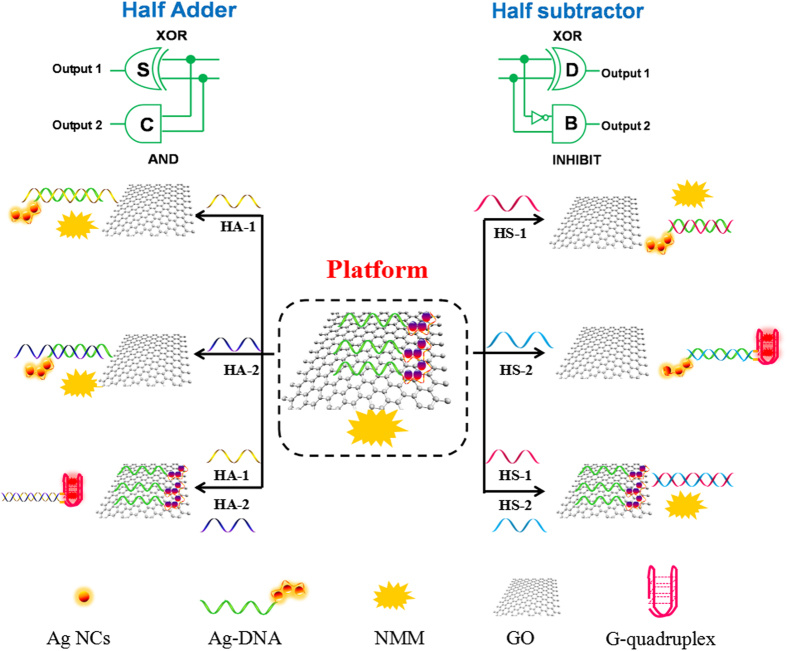
Operation mechanisms of the developed half adder and half subtractor along with the corresponding logic circuits.

**Figure 2:**
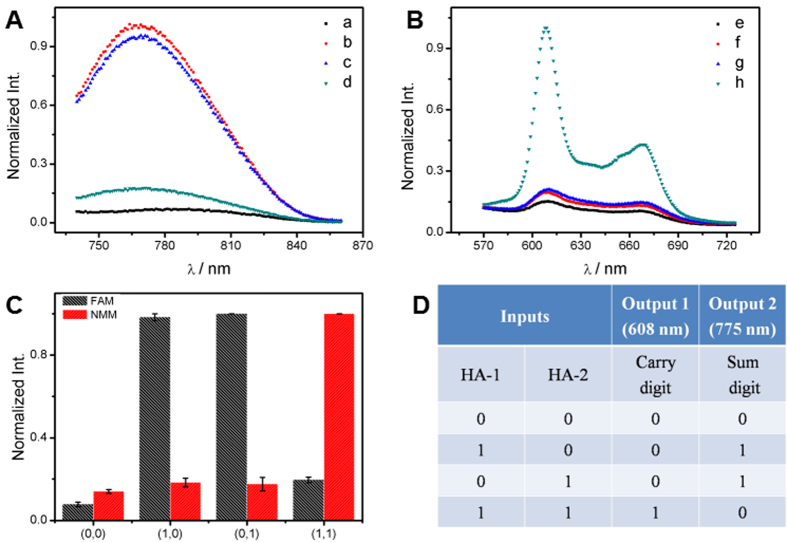
(**A,B**) The output signals of AgNCs and NMM for the half adder operation triggered by the various inputs: No input (a,e); in the presence of HA-1 (600 nM; b,f), HA-2 (600 nM; c,g), and HA-1/HA-2 (1:1; d,h). (**C**) The normalized fluorescence intensity of NMM at 608 nm and AgNs at 775 nm as a function of the various inputs. (**D**) The truth table of the HA logic operation.

The HA logic circuit is schematically shown in Fig. 1. In the initial state, Ag-DNA bound on the surface of GO resulted in the low fluorescence intensity of AgNCs (curve (a) in Fig. 2A) and NMM (curve (e) in Fig. 2B). In the presence of any one input, HA-1 or HA-2, its hybridization with Ag-DNA generated ds-DNA leading to the separation of Ag-DNA from GO and the enhancement of AgNCs fluorescence, corresponding to Fig. 2A(b,c). On the other hand, changes in the fluorescence intensity of NMM were negligible due to the fact that no G-quadruplex was produced in the Ag-DNA/HA-1 (f) and Ag-DNA/HA-2 (g) as seen from Fig. 2B. In the coexistence of HA-1 and HA-2, the hybridization between HA-1 and HA-2 took place prior to their respective interactions with Ag-DNA. Therefore the Ag-DNA was still anchored on GO surface and emitted low fluorescence, Fig. 2A(d). The complexation between the two inputs facilitated the formation of G-quadruplex between G-rich splits GGGT at the 5′-terminal of HA-1 and a G-rich sequence (GGGTTTTGGGTTTTGGG) at the 3′-terminal of HA-2. Consequently, the formed G-quadruplex structure dramatically increased the fluorescence signal of NMM, Fig. 2B(h). The results shown in Fig. S5 clearly demonstrated the above DNA interactions by the native polyacrylamide gel electrophoresis (PAGE). The presence and absence of inputs are defined as “1” and “0”; the normalized fluorescence intensities above or below the threshold value of 0.35 to AgNCs and NMM correspond to output “1” or “0”, respectively. The definition is available for all the logic gates (half adder, half subtractor, multiplexer, and demultiplexer) in this work. The normalized fluorescence intensities of NMM (at 608 nm) and AgNCs (at 775 nm) are plotted as column bar in Fig. 2C, and the corresponding truth table is shown in Fig. 2D. Obviously, both NMM and AgNCs related logic circuits fit the characteristics of the AND and XOR logic gates, which code for the CARYY (C) and SUM (S) digits of HA, respectively. The logic circuits could be performed in parallel and triggered by the same set of inputs. Thus a half adder was fabricated reasonably.

**Figure 3:**
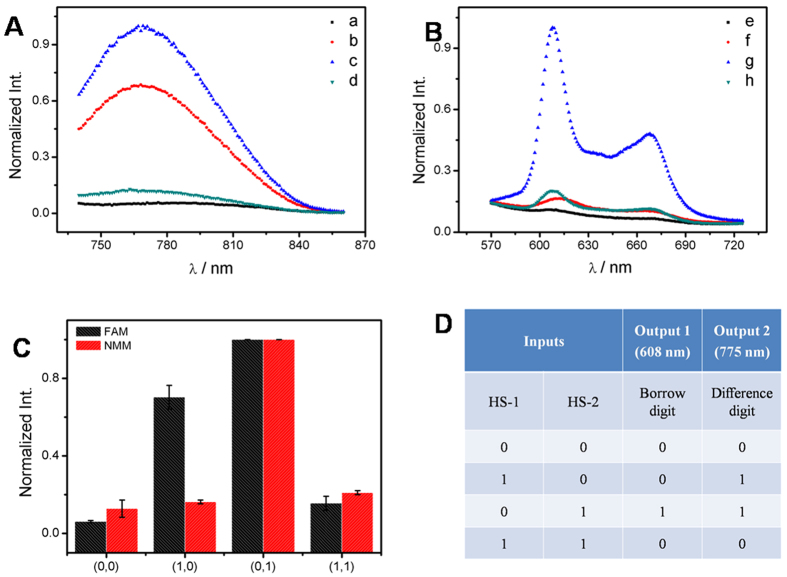
The output signals of AgNCs (**A**) and NMM (**B**) for the half subtractor operation triggered by the various inputs. No input (a,e); in the presence of HS-1 (500 nM; b,f); HS-2 (550 nM; c,g); HS-1/HS-2 (1:1.1; d,h). (**C**) The normalized fluorescence intensity of NMM at 608 nm and AgNCs at 775 nm as a function of the various inputs (HS-1 and HS-2). (**D**) The truth table of the HS logic operation.

A half subtractor operation was implemented by two new inputs (HS-1 and HS-2) on the same DNA/GO platform (Fig. 1). For the HS arithmetic logic function, the parallel XOR and INHIBIT logic gates performed the DIFFERENCE and the BORROW digits, respectively. As the XOR gate is also required in half subtractor, hence, the inputs of HS were designed by the similar strategy in HA and the fluorescence of AgNCs was employed as the output signal for the XOR gate. In the presence of either input, the HS-1 or HS-2, the Ag-DNA could be released from GO by forming the duplex of Ag-DNA/HS-1 or Ag-DNA/HS-2. In the coexistence of HS-1 and HS-2, HS-1 and HS-2 would hybridize preferentially and the Ag-DNA was still trapped on the GO surface with low fluorescence (Fig. 3A(d)). Only if the input was added individually, enhanced fluorescence of AgNCs could be observed, Fig. 3A(b,c). The DNA interactions were confirmed by the PAGE (Fig. S7). Meanwhile, INHIBIT logic operation was implemented using NMM fluorescence as output signal. The HS-2 contains the G-rich sequence, which can form the G-quadruplex structure, at the 3′-terminus. Due to the C-rich sequence at its 5′-terminus, HS-1 can inhibit the G-quadruplex formation of HS-2 when the duplex of HS-1/HS-2 is formed. Hence, the system showed a strong fluorescence output signal of NMM when only HS-2 was added (Fig. 3B(g)), and a low NMM fluorescence signal was observed under other circumstance, Fig. 3B(e,f,h). The normalized fluorescence signals of NMM (at 608 nm) and AgNCs (at 775 nm) are plotted as the column bar in Fig. 3C. The developed AgNCs-related XOR gate and NMM-related INHIBIT gate correspond to the DIFFERENCE and the BORROW digits of HS, respectively. The obtained results of the truth table obviously fulfills the requirement for the HS operation (Fig. 3D).

### Operation of Nonarithmetic Functions

Considering that the importance of nonarithmetic functions in molecular computing, multiplexer (MUX) and demultiplexer (DEMUX) were constructed on the same DNA/GO platform. The MUX can transmit multiple data streams into a single output channel^30^. Oppositely, the DEMUX is able to transmit a single data line into multiple data streams^32^. The MUX and the DEMUX play an important role in telecommunication and signal processing systems^33^,^34^. Up till now, it is still a challenge to fabricate the complex logic device in the molecular level^35^. Herein, we present a 2:1 MUX and a 1:2 DEMUX based on the synergistic action of GO and DNA.

**Figure 4:**
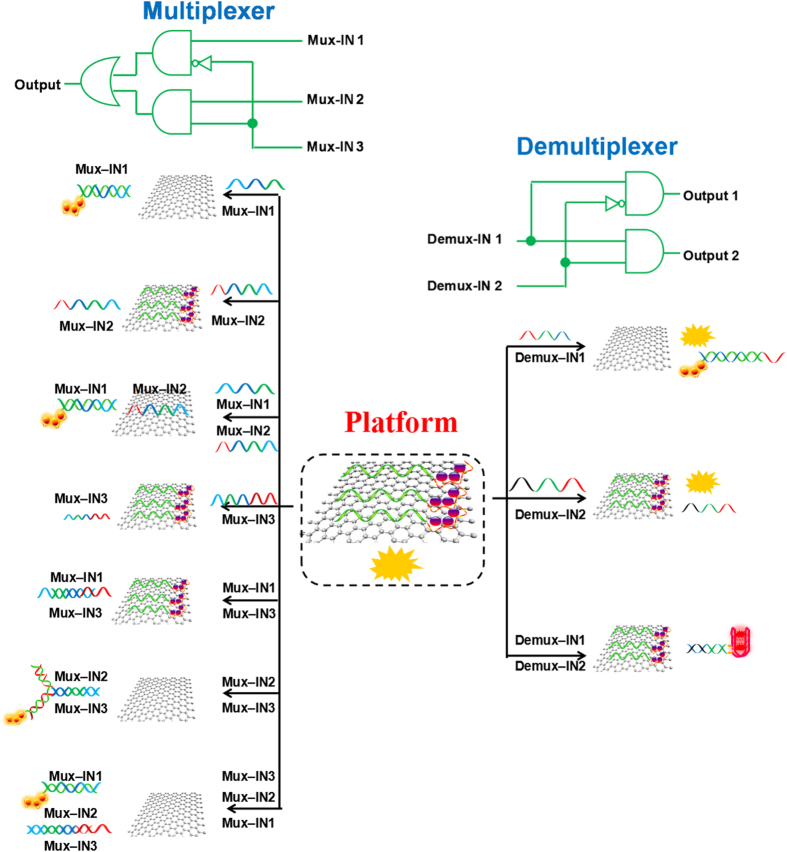
The operation mechanism of the developed 1:2 demultiplexer and 2:1 multiplexer with the corresponding logic circuits.

**Figure 5:**
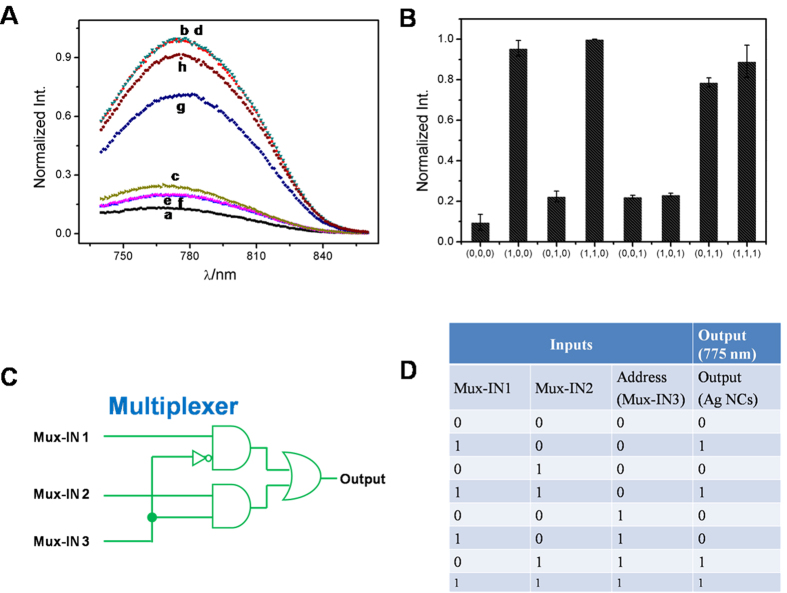
(**A**) The output signals of AgNCs for the 2:1 MUX triggered by the various inputs. No input (a); in the presence of Mux-IN1 (b); Mux-IN2 (c); Mux-IN1/Mux-IN2 (d); Mux-IN3 (e); Mux-IN3/Mux-IN1 (f); Mux-IN3/Mux-IN2 (g); Mux-IN3/Mux-IN1/Mux- IN2 (h). (**B**) The normalized fluorescence intensity of AgNCs at 775 nm as a function of the various inputs. The corresponding logic circuit (**C**) and truth table (**D**) of the MUX logic gate.

The 2:1 MUX is a communication device which can transmit two data inputs into a single output under the function of one address input. As shown in Fig. 4, the three inputs of the MUX are defined as Mux-IN1 (the data input), Mux-IN2 (the data input) and Mux-IN3 (the address input), and the fluorescence of AgNCs is set as the output signal. In the absence of the Mux-IN3, the output would report the state of the Mux-IN1; whereas in the presence of Mux-IN3, the state of Mux-IN2 would be a mirror of the output. Thus, we first discuss the situation in the absence of Mux-IN3. Without any input, the Ag-DNA was stuck on GO, which induced low fluorescence of AgNCs, Fig. 5A(a). When adding Mux-IN1 into the system, the hybridization of Mux-IN1 and Ag-DNA would strip the Ag-DNA from the GO surface leading to strong fluorescence of AgNCs, Fig. 5A(b). Since the Mux-IN2 could not hybridize with the Ag-DNA, weak emission from AgNCs was observed, Fig. 5A(c). Upon simultaneous addition of two inputs, Mux-IN1 and Mux-IN2, the interaction between Mux-IN1 and Ag-DNA was designed to be superior to the hybridization of Mux-IN1/Mux-IN2, which resulted in Ag-DNA/Mux-IN1 complex and free Mux-IN2. The formed Ag-DNA/Mux-IN1 complex gave rise to the separation of Ag-DNA from the GO surface and produced strong fluorescence of AgNCs, Fig. 5A(d).

In the presence of address input (Mux-IN3), the output would report the state of the Mux-IN2. In the absence of any data inputs, weak fluorescence of AgNCs was recorded in the system containing Mux-IN3, Fig. 5A(e), because the Mux-IN3 could not hybridize with the Ag-DNA. When the Mux-IN1 was added into the system containing Mux-IN3, the hybridization between Mux-IN1 and Mux-IN3 occurred preferentially instead of interaction between Mux-IN1 and Ag-DNA. Thus the Ag-DNA, which was absorbed on the surface of GO, emitted weak fluorescence, Fig. 5A(f). When the Mux-IN2 was introduced into the system with Mux-IN3, a three-component structure generated among Mux-IN2, Mux-IN3 and Ag-DNA. The three-component structure liberated the Ag-DNA from GO, yielding strong AgNCs fluorescence, Fig. 5A(g). In the last case of simultaneous addition of two inputs, the Ag-DNA/Mux-IN1 and Mux-IN2/Mux-IN3 complexes were the main products in the system, and a strong AgNCs fluorescence signal could be observed due to the release of Ag-DNA, Fig. 5A(h). The DNA interactions mentioned in the strategy were validated by PAGE analysis (Fig. S8). The normalized fluorescence response of AgNCs at 775 nm was recorded as the output signal and the corresponding column bars are shown in Fig. 5B. The truth table (Fig. 5D) produced by the fluorescence results fully meets the requirements in a 2:1 MUX operation. The Fig. 5C shows the corresponding logic circuit.

**Figure 6:**
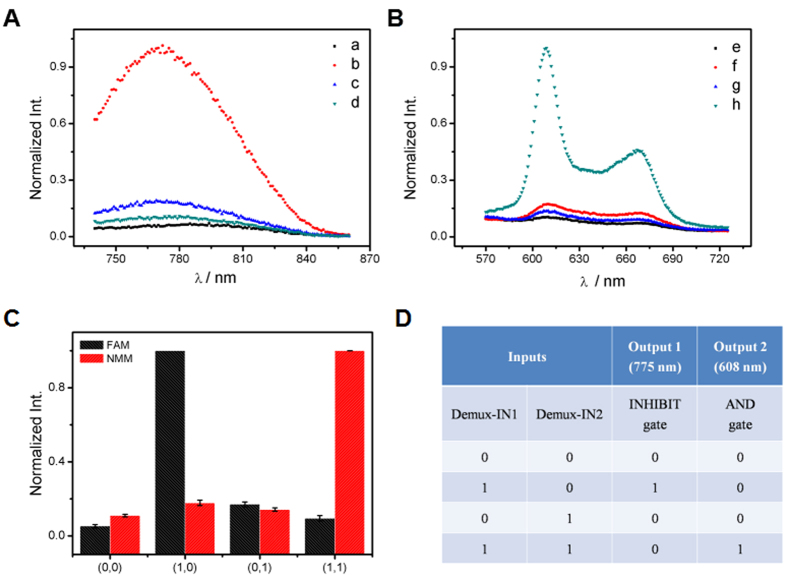
The output signals of AgNCs (**A**) and NMM (**B**) for the DEMUX logic operation triggered by the various inputs: No input (a,e); in the presence of Demux-IN1 (b,f); Demux-IN2 (c,g); Demux-IN1/Demux-IN2 (d,h). (**C**) The normalized fluorescence intensity of AgNCs at 775 nm and NMM at 608 nm as a function of the various inputs. (**C**) The truth table of the DEMUX logic gate.

As the opposite logic circuit of 2:1 MUX, the 1:2 DEMUX can transmit one compress signal into two data streams controlled by an additional address input^36^. Herein, 1:2 DEMUX was subsequently fabricated with the same DNA/GO platform. To implement the function of 1:2 DEMUX, two inputs (data input and address input) and two outputs (signals) were required. The AgNCs and NMM were selected as the signal reports. The principle of the 1:2 DEMUX is depicted in Fig. 4. The Demux-IN1 acted as the data input, which could partly hybridize with Ag-DNA and allow the Ag-DNA to release from GO. The hybridization between Demux-IN1 and Ag-DNA was confirmed by the strong AgNCs fluorescence response, Fig. 6A(b). The Demux-IN2, which is defined as the address input, was designed to be unresponsive to the Ag-DNA. Thus weak fluorescence signal was collected in the system containing Demux-IN2, Fig. 6A(c). In the coexistence of both inputs, Demux-IN1 preferred to hybridize with Demux-IN2 to form Demux-IN1/Demux-IN2 complex rather than with Ag-DNA to form Ag-DNA/Demux-IN1. Thus, a low fluorescence signal of AgNCs was generated, Fig. 6A(d). In addition, the Demux-IN1 contains G-rich segment (GGGTTTTGGGTTTTGGG) at the 3′-terminal, and the Demux-IN2 contains GGGT at the 5′-terminal. The hybridization between Demux-IN1 and Demux-IN2 promoted formation of G-quadruplex structure, which conspicuously enhanced the NMM fluorescence, Fig. 6B(h). In other cases, the low NMM fluorescence signal was monitored due to the lack of G-quadruplex structure, Fig. 6B(e,f,g). The interactions among all the DNA strands associated with the developed 1:2 DEMUX were demonstrated by PAGE analysis (Fig. S9). The normalized fluorescence response of AgNCs at 775 nm was recorded as the output signal and the corresponding column bars are shown in Fig. 6C. The truth table (Fig. 6D) of a 1:2 DEMUX operation was obtained according to the fluorescence results.

## Conclusions

A series of complex logic gates have been creatively constructed on a label-free, enzyme-free and universal platform to perform arithmetic functions and nonarithmetic information processing, including HA, HS, MUX and DEMUX functions. The platform was developed based on graphene oxide and DNA-templated silver nanoclusters. All the logic circuits shared the same threshold set-point and the same mode of signal outputs. Furthermore, in each logic operation, dual outputs were generated in parallel and stimulated by the same set of inputs. The cost-effective and universal platform is more capable of meeting the needs of future practical applications. For example, the biocompatible platform can be applied in the biomedical field for disease diagnosis and therapy. Moreover, the advanced molecular system based on near-infrared fluorescence of AgNCs and GO has great potentials in smart bioimaging and disease diagnosis attributed to the biocompatibility of DNA.

## Methods

### Materials and Instrumentations

Sequences of DNA strands are listed in Table S1. DNA oligonucleotides were purchased from Shanghai Sangon Biotechnology Co (Shanghai, China). Stock solutions were prepared by dissolving appropriate amount of DNA oligonucleotide in pure water and the concentrations of the solutions were determined by measuring UV-visible absorption at 260 nm. N-methylmesoporphyrin IX (NMM) was purchased from Porphyrin Products (Logan, UT, USA). All the other analytical pure chemicals were obtained from Aladin (Shanghai, China) and used as received without further purification. GO was prepared according to a modified Hummer’s method^28^. Milli-Q water (18.2 MΩ) was used throughout. UV-vis absorption measurements were performed on a Cary 500 Scan UV/Vis/NIR Spectrophotometer (Varian, USA). Fluorescence measurements were carried out on a Fluoromax-4 spectrofluorometer (Horiba JobinYvon, Inc., NJ, USA). The fluorescence spectra were recorded at room temperature by irradiating AgNCs at 714 nm and NMM at 399 nm, respectively.

### Preparation of DNA-Stabilized AgNCs

The AgNCs were prepared according to the reported protocol^25^. Shortly, 10 μL of the DNA template (100 μM) was mixed with 70 μL of phosphate buffer (10 mM, pH 7) and the mixture was heated at 90 °C for 10 min. The solution was afterwards annealed to room temperature. To this solution, 10 μL of freshly prepared AgNO_3_ aqueous solution (1.6 mM) was added, followed by virous shaking of the solution for 30 s. After incubation for 20 min, 10 μL of freshly prepared NaBH_4_ aqueous solution (0.8 mM) was added to the solution, followed by vigorous shaking of the mixture for 1 min. The mixture was kept in dark at room temperature and allowed to perform the reaction for 12 h. The obtained AgNCs were kept in 4 °C for use without any further purifications or pre-treatments. The excitation and emission spectra of the AgNCs are presented in Fig. S1.

### Logic Gate Operations

The DNA solutions were heated at 90 °C for 10 min and then gradually cooled down to room temperature before conducting further experiments. The GO (8 μg/mL) and Ag-DNA (50 nM) were added into 10 mM phosphate buffer containing 20 mM CH_3_COOK and 5 mM Mg(CH_3_COO)_2_ (pH 7). The NMM (1 μM) was afterwards added into the GO/Ag-DNA complex to form the platform. The various inputs were added into the platform and incubated for 30 min. The fluorescence responses of the system were recorded. The quantity of GO and all the inputs were optimized by monitoring the variation of fluorescence intensity of the system. For the implementation of the arithmetic functions, the fluorescence responses of the system were recorded after mixing various inputs with the platform for 30 min. The incubation time was optimized according to the time-dependent restoration of AgNCs fluorescence after adding the input. The details of the optimal experiments can be found in Figs S2–S4 and S6 in supporting information.

### Native polyacrylamide gel electrophoresis

Polyacrylamide gel (12%) was prepared with 1×Tris-borate-EDTA buffer (89 mM Tris, 89 mM boric acid, 2 mM EDTA, pH 8.3). Each sample was prepared with 1×Tris-borate-EDTA buffer containing 12 mM Mg^2+^, and the concentration of each DNA strand was 2 μM. The sample solution was heated at 90 °C for 10 min and then annealed slowly to room temperature. 20 μL of each sample was mixed with 2 μL of Gel-Dye Super Buffer Mix before loading into the gel. The gel was run under a constant voltage of 100 V over a period of about 2.5 h. Photographs were taken under UV light by using a fluorescence imaging system (Vilber Lourmat, Marne laVallee, France).

## Additional Information

**How to cite this article**: Wang, K. *et al*. Implementation of Arithmetic and Nonarithmetic Functions on a Label-free and DNA-based Platform. *Sci. Rep.* **6**, 34810; doi: 10.1038/srep34810 (2016).

## Supplementary Material

Supplementary Information
