# Supplementary material for: Implementation of Arithmetic and Nonarithmetic Functions on a Label-free and DNA-based Platform
Source: Sci Rep. 2016 Oct 7;6:34810. doi: 10.1038/srep34810 (PMC5054380; doi:10.1038/srep34810)
Supplement: Supplementary Information [file srep34810-s1.doc]

Supporting Information

**Implementation of Arithmetic and Nonarithmetic Functions on a Label-free and DNA-based Platform**

Kun Wang,^1^ Mengqi He^2^, Jin Wang^1^, Ronghuan He,^*1^ Jianhua Wang^*2^

^1^ Department of Chemistry, College of Sciences, Northeastern University, Shenyang 110819, China

^2^ Research Center for Analytical Sciences, College of Sciences, Northeastern University,

Shenyang 110819, China

* Corresponding authors. E-mail: herh@mail.neu.eud.cn (R. He); jianhuajrz@mail.neu.eud.cn (J. Wang)

**Table S1. DNA sequences used in this work**

|  | Strands | Sequence (from 5’-terminal to 3’terminal) |
| --- | --- | --- |
| Platform | Ag-DNA | CCCACCCACCCTCCCATTT TAACG TGTGT TTGCA CTATG CTTTCA CTGC TTA |
| Half adder | HA-1 | TAATATATGAAAGCAGATTGCAAAACCAAGTAGCATTATAGGGTT TTGGGTTTTGGG |
|  | HA-2 | GGGTATAATGCTACTTGGTAAAGCAATCTGCAAACATATATTA |
| Half subtractor | HS-1 | TAATATATGAAAGCAGATTGCAAACCAAGTAGCCCCAAAACCCAAAACCC |
|  | HS-2 | GGGTTTTGGGTTTTGGGTTTTGGGGCTACTTGGAAAGCAATCTGCAAACATATATTA |
| Multiplexer | Mux-IN1 | AAACCCCCGCATAGTGCCCCCACACGTTA |
|  | Mux-IN2 | GCAAACAGGCCCTGTAAACCCCCGCATTCTGCCCCCACA |
|  | Mux-IN3 | TGTGGGGGCAGAATGCGGGGGTTTACAGGGCCCACGTTA |
| Demultiplexer | Demux-IN1 | TAAGCAGTGTTTGCATAGTGCTTTCACACGTTAAGGGTTTTGGGTTTTGGG |
|  | Demux-IN2 | GGGTTAACGTGTGAAAGCACTATGCAAACACTGCTTA |

For the purpose of developing higher-order logic gates with available DNA sequences, the designed DNA sequences were first mimicked on the website at http://mfold.rna.albany.edu/?q=DINAMelt/Twostate- melting and they were then modified according to the experimental results. The above procedures were repeated until the available DNA sequences were obtained.


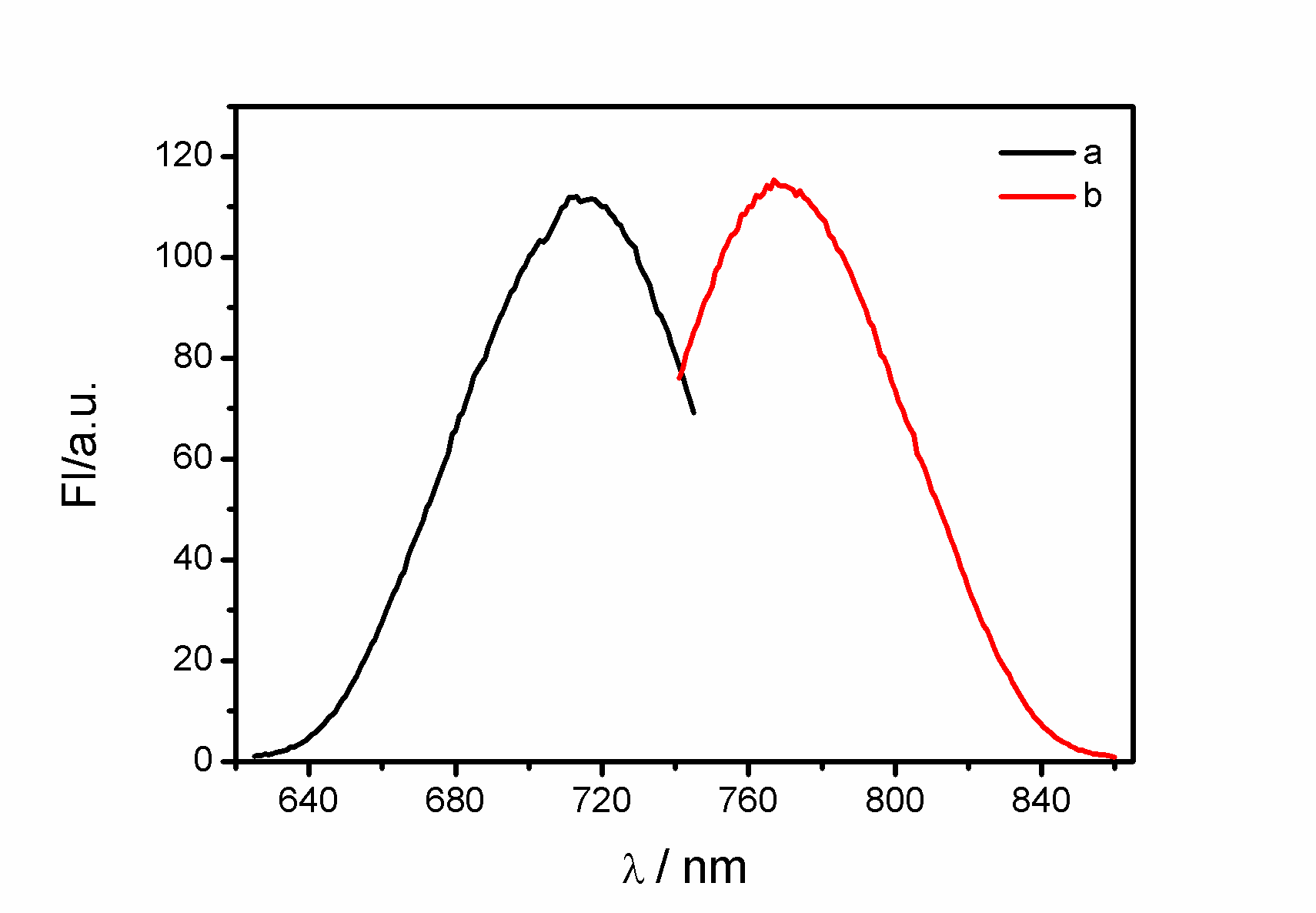


Figure S1**.** Excitation (a) and emission (b) spectra of the red-emitting AgNCs stabilized by Ag-DNA.


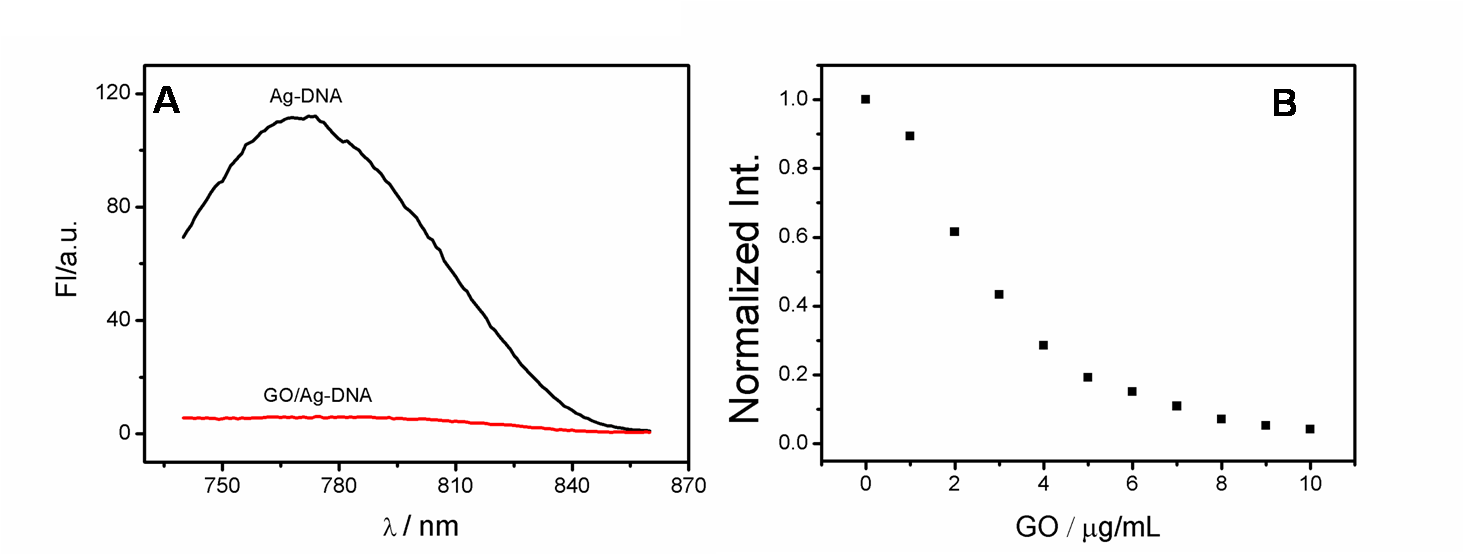


Figure S2. (A) AgNCs fluorescence response before and after addition of GO. (B) AgNCs fluorescence response in the presence of various concentrations of GO.

As shown in Figure S2 (A), the fluorescence intensity of AgNCs was significantly quenched once Ag-DNA was bound on the GO surface via noncovalent π-π stacking interaction. The GO acted as a quencher and discriminator and its concentration affected obviously on the performance of the logic gate. At a low concentration of GO, the fluorescence of AgNCs could not be effectively quenched, leading to a strong signal background. On the contrary, the fluorescence of AgNCs could not be effectively recovered at a too high concentration of GO. Herein, by monitoring the variations of fluorescence intensity of AgNCs as a function of GO concentration at 775 nm, the optimal concentration of GO could be derived. It is seen that a decrease of fluorescence intensity of AgNCs was observed along with the increase of the GO concentration and a plateau was reached at 8 μg/ml. For further experiments, 8 μg/ml of GO was used.


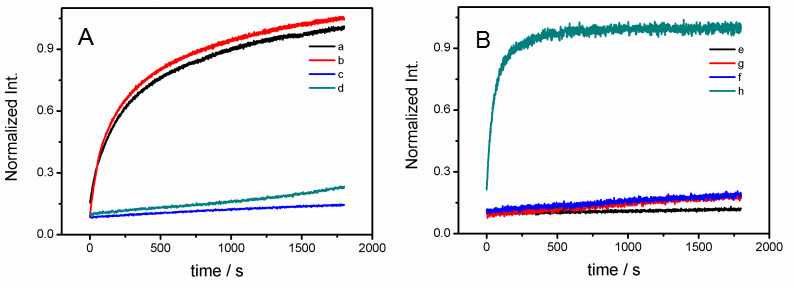


Figure S3. The time-dependent fluorescence intensity change of the AgNCs (A) and NMM (B) for the half adder operation triggered by the various inputs: No input (a, e); in the presence of HA-1 (600nM; b, f), HA-2 (600nM; c, g), and HA-1/HA-2 (1:1; d, h).

After the addition of the input, the fluorescence intensity of the AgNCs and NMM increases with time. The incubation time is then determined after the fluorescence signal reaches a plateau (Fig. S3). Hence, the incubation time of 30 min was used for the following experiments.


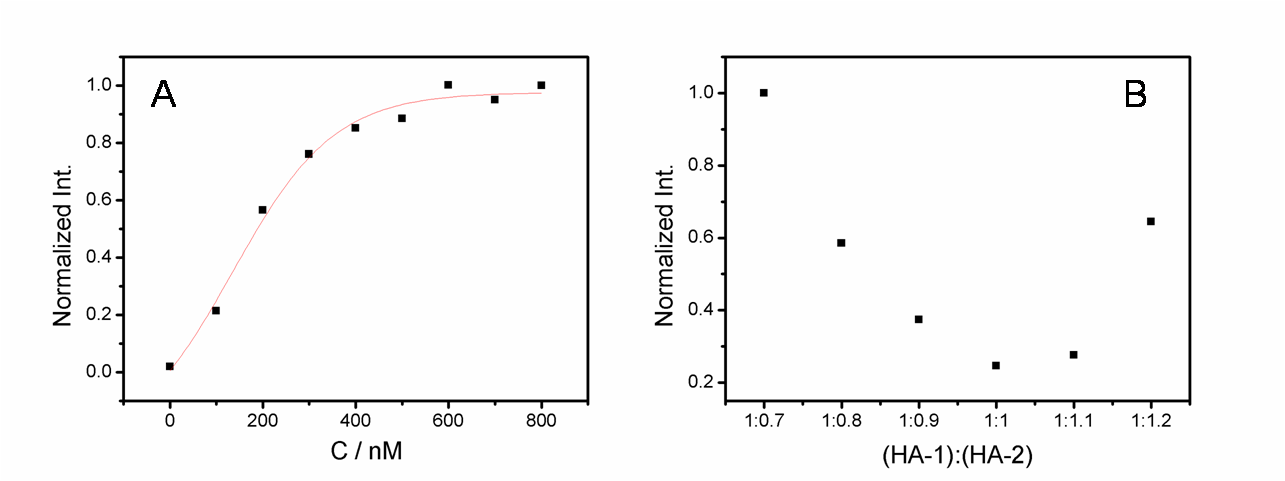


Figure S4. (A) The AgNCs fluorescence restoration of GO/Ag-DNA as a function of the HA-1 concentration for the developed half-adder. (B) The fluorescence response of AgNCs as a function of different concentration ratio of the two inputs, HA-1 and HA-2.

As shown in Figure S4 (A), the AgNCs fluorescence intensity of GO/Ag-DNA increased with the increase in the concentration of HA-1 and reached a plateau at a concentration of up to 600 nM. Thus, 600 nM was selected for the ensuing experiments. For the AgNCs-related XOR logic gate of HA function, the system presented a low output in the coexistence of two inputs. To get an optimal low output in this case, the concentration ratio of the inputs was investigated to reduce the signal background. As learned from Figure S4 (B), a minimum AgNCs fluorescence intensity was observed at a HA-1/HA-2 ratio of 1:1. Therefore, the optimal concentration was found at 600 nM for both HA-1 and HA-2.


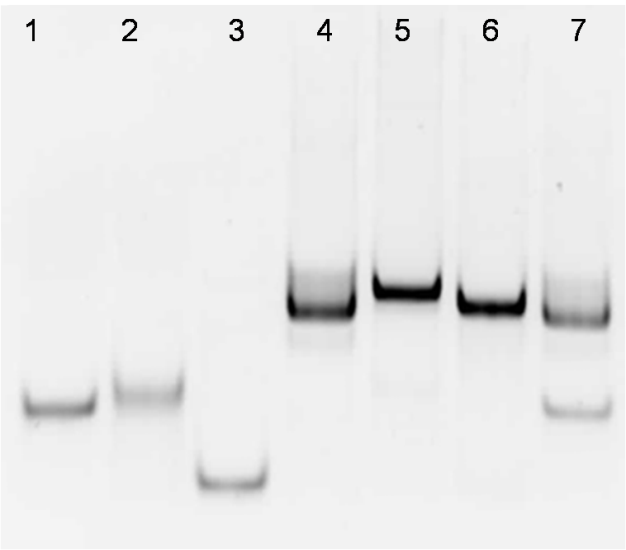


Figure S5. PAGE analysis of Ag-DNA (1), HA-1 (2), HA-2 (3), HA-1/HA-2 (4), Ag-DNA/HA-1 (5), Ag-DNA/HA-2 (6), and Ag-DNA/HA-1/HA-2 (7).

Figure S5 (A) shows the polyacrylamide gel analysis of the interaction between different DNA strands used in HA logic operation. Lane 1 to Lane 3 shows the DNA bands of Ag-DNA, HA-1, and HA-2, respectively. In the presence of any two DNA strands, (HA-1 and HA-2), (Ag-DNA and HA-1) or (Ag-DNA and HA-2), new bands appeared from Lane 4 to Lane 6. The results indicate the formation of duplexes of HA-1/HA-2, Ag-DNA/HA-1 and Ag-DNA/HA-2. In the presence of Ag-DNA, HA-1 and HA-2, one band appeared at the level of Ag-DNA, and another band was found at the level of duplex HA-1/HA-2 as shown in Lane 7, which indicated the priority of hybridization between HA-1 and HA-2 over their respective interactions with Ag-DNA. The results of PAGE and fluorescence experiments were consistent with each other.


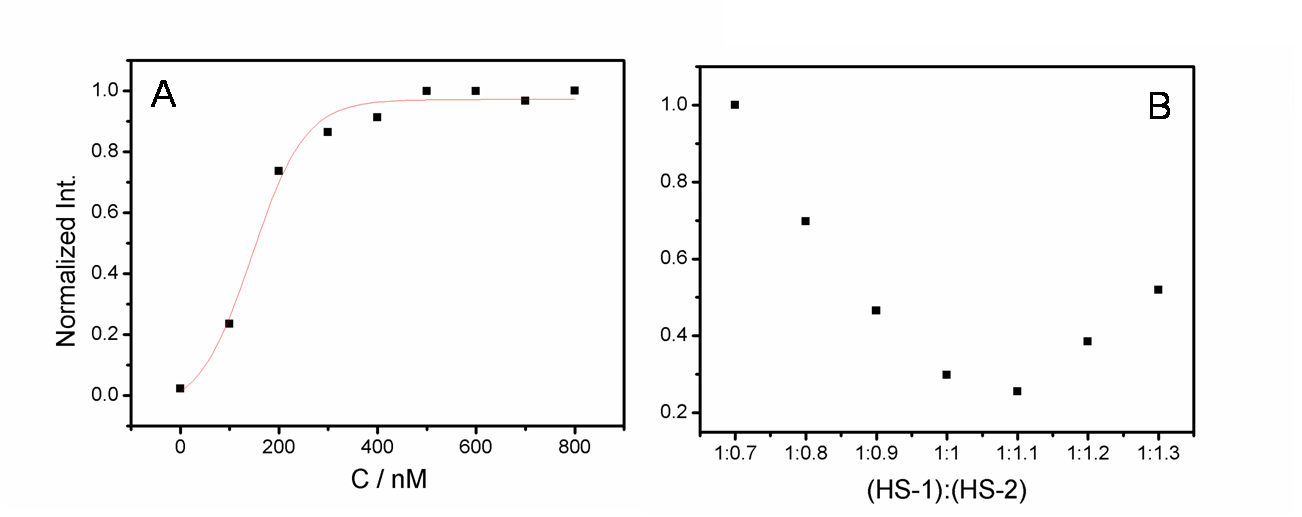


Figure S6. (A) The AgNCs fluorescence restoration of GO/Ag-DNA as a function of the HS-1 concentration for the developed half-substractor. (B) The fluorescence response of AgNCs as a function of different concentration ratio of the two inputs, HS-1 and HS-2.

As shown in Figure S6 (A), the AgNCs fluorescence intensity of GO/Ag-DNA increased with the increase in concentration of HS-1 and reached a plateau up to 500 nM. For the AgNCs-related XOR logic gate of HS function, the system presented a low output in the coexistence of the two inputs. To get an optimal low output in this case, the concentration ratio of the inputs was investigated to minimize the signal background. It is seen from figure S6 (B) that the AgNCs fluorescence intensity reached a minimum at a HS-1/HS-2 ratio of 1:1.1. Therefore, the optimal concentrations of 500 nM and 550 nM were used for the two inputs, HS-1 and HS-2, respectively.


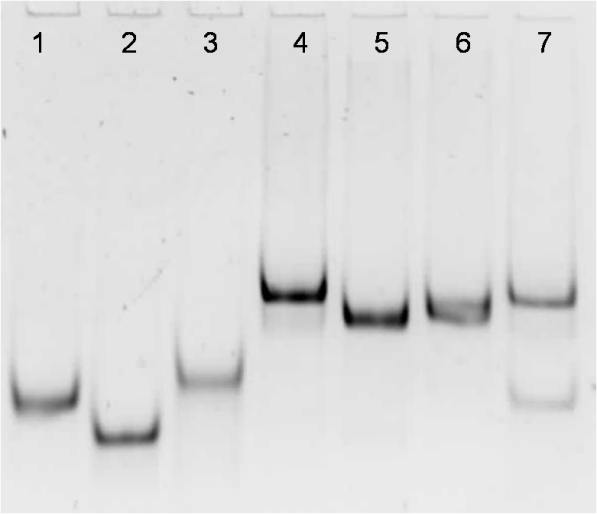


Figure S7. PAGE analysis of Ag-DNA (1), HS-1 (2), HS-2 (3), HS-1/HS-2 (4), Ag-DNA/HS-1 (5), Ag-DNA/HS-2 (6), and Ag-DNA/HS-1/HS-2 (7).

PAGE experiments were also performed to further identify the DNA interaction in HS logic operation, Figure S7. Lanes 1 to 3 show the DNA bands of Ag-DNA, HS-1 and HS-2, respectively. The new bands shown in Lane 4, Lane 5 and Lane 6 indicated the hybridization between HS-1 and HS-2, Ag-DNA and HS-1, Ag-DNA and HS-2, respectively. In the presence of Ag-DNA, HS-1 and HS-2, two separated bands showed in Lane 7. One appeared at the level of Ag-DNA, and another at the level of duplex HS-1/HS-2, suggesting that the hybridization between HS-1 and HS-2 was favored over their hybridization with Ag-DNA.


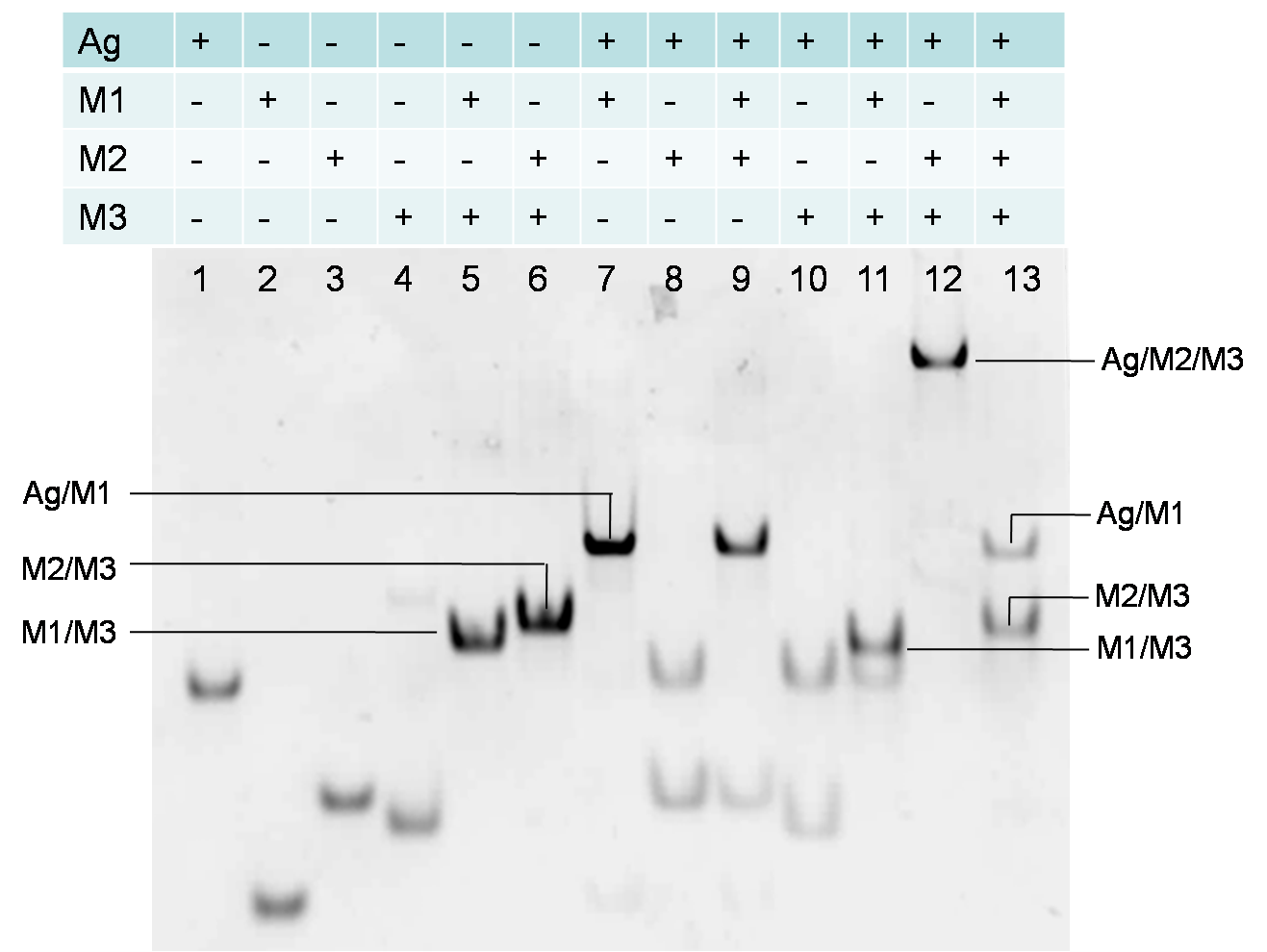
Figure S8. Native polyacrylamide gel analysis of the interactions among Ag-DNA, Mux-IN1, Mux-IN2, and Mux-IN3 (Address input: A). DNA strands Ag-DNA, Mux-IN1, Mux-IN2, and Mux-IN3 are abbreviated as Ag, M1, M2, and M3, respectively. The sample in each lane and the identities of the main bands are indicated above and at the sides of the gel image, respectively.

PAGE analysis of the interactions among the four DNA strands (Ag-DNA and three inputs) in the 2:1 multiplexer was achieved through band comparison. The bands in lanes 1, 2, 3, and 4 correspond to Ag-DNA, Mux-IN1, Mux-IN2, and Mux-IN3, respectively. The new bands in lanes 5, 6 and 7 indicate the formation of Mux-IN1/Mux-IN3, Mux-IN2/Mux-IN3 and Ag-DNA/Mux-IN1 complexes, respectively. On the contrast, the interaction between Ag-DNA and Mux-IN2 did not happen via comparing lane 8 with lane 1 and lane 3, and nor did Ag-DNA and Mux-IN3 in lane 10. In lane 9, the mixture of Ag-DNA, Mux-IN1 and Mux-IN2 generated a clear band ascribed to Ag-DNA/Mux-IN1 complex and a weakly visible Mux-IN2 band. In lane 11, the mixture of Ag-DNA, Mux-IN1 and Mux-IN3 produced two clear bands, which belong to Ag-DNA and Mux-IN1/Mux-IN3 complex, respectively, through comparison with lanes 1 and 5. In lane 12, the clear band was ascribed to three-component Ag-DNA/Mux-IN2/Mux-IN3 complex in comparison with lane 1 and lane 6. In lane 13, two clear bands corresponding to Ag-DNA/Mux-IN1 and Mux-IN2/Mux-IN3 complexes appeared in the presence of all three inputs.


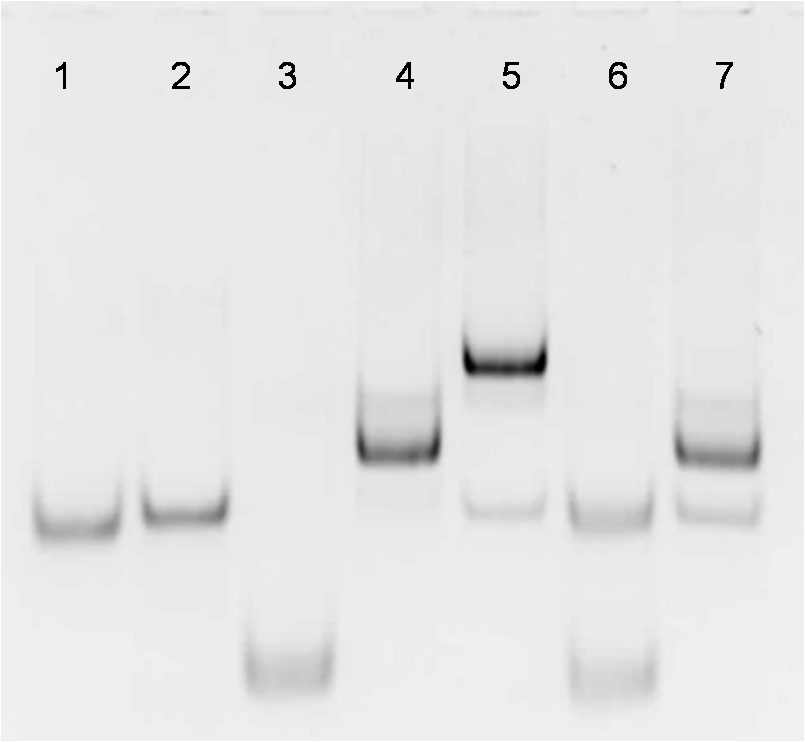


Figure S9. PAGE analysis of Ag-DNA (1), Demux-IN1 (2), Demux-IN2 (3), Demux-IN1/ Demux-IN2 (4), Ag-DNA/ Demux-IN1 (5), Ag-DNA/ Demux-IN2 (6), Ag-DNA/ Demux-IN1/ Demux-IN2 (7)

PAGE experiments were conducted to validate the DNA interactions in DEMUX logic operation. From Lane 1 to Lane 3 in Figure S9, the bands indicate the individual ss-DNAs of Ag-DNA, Demux-IN1 and Demux-IN2 in sequence. The new bands shown in Lane 4 and Lane 5 indicate the hybridization between Demux-IN1 and Demux-IN2, Ag-DNA and Demux-IN1, respectively. The separated two bands shown in Lane 6 suggest that Ag-DNA did not hybridize with Demux-IN2. In the presence of Ag-DNA and IN1, a new band appeared at a different position in Lane 4, indicating the formation of duplex of F-DNA/IN1. In the coexistence of Ag-DNA, Demux-IN1 and Demux-IN2, two bands were found from Lane 6. One band appeared at the position similar to that of Ag-DNA, another appeared at a position similar to that of Demux-IN1/Demux-IN2. The PAGE result indicates that the DNA interactions occurred as expected.
